# Case Report: Stepwise noninvasive diagnosis of Takotsubo cardiomyopathy in an elderly patient–From ECG clues to echocardiographic and CTA confirmation

**DOI:** 10.3389/fcvm.2025.1608992

**Published:** 2025-06-04

**Authors:** Yanling Teng, Xingxing Sun, Minglang Wang, Ziyang Wang, Yilian Wang

**Affiliations:** ^1^ Department of Cardiology, The First People’s Hospital of Lianyungang, The First Affiliated Hospital of Kangda College of Nanjing Medical University, Lianyungang, China; ^2^ Department of Cardiology, The Second People’s Hospital of Lianyungang, Affiliated to Kangda College of Nanjing Medical University, Lianyungang, China

**Keywords:** Takotsubo cardiomyopathy, noninvasive diagnosis, elderly, ECG, coronary CTA

## Abstract

**Background:**

Takotsubo cardiomyopathy (TTC) is frequently misdiagnosed as acute coronary syndrome in elderly patients. This case demonstrates how ECG findings facilitate a noninvasive diagnostic algorithm for TTC, validated by echocardiography and coronary computed tomography angiography (CCTA).

**Case summary:**

An 88-year-old woman presented with chest tightness and dyspnea after emotional stress (bereavement). Initial ECGs showed the concurrent appearance of ST-segment elevations in anterior (V3-V5) and inferior leads (II, III, aVF), suggesting apical injury and the diagnosis of TTC. Bedside echocardiography revealed apical akinesis with preserved basal contraction (LVEF 35%), while CCTA ruled out obstructive disease. Supportive therapy led to symptom resolution. At 1-year follow-up, LVEF recovered to 61% with normalized ECG.

**Conclusion:**

This case highlights ECG's pivotal role in suspecting TTC, enabling a Noninvasive diagnostic approach (echocardiography + CCTA) for elderly patients.

## Introduction

Takotsubo cardiomyopathy (TTC), first described in Japan in 1990, represents a fascinating paradox in cardiovascular medicine—an acute reversible cardiomyopathy mimicking myocardial infarction in the absence of obstructive coronary artery disease (CAD) (1). While its classic apical ballooning variant accounts for 81.7% of cases, diagnostic accuracy remains limited in elderly populations, where comorbidities often mask characteristic features (2).

Box 1 **Timeline.**

**Table:** 

3-Days before admission	The patient experienced chest tightness and dyspnea following emotional stress (bereavement). Electrocardiograms were obtained on consecutive days from a local community clinic.
Day 0	An 88-year-old female patient, experiencing chest tightness and shortness of breath for three days, was admitted to the hospital. An electrocardiogram (ECG) was performed to take a suspected diagnosis of Takotsubo Syndrome (TTS).
Day 0	Echocardiography was subsequently performed, revealing significant attenuation or even absence of systolic function at left ventricular apex, the left ventricular ejection fraction (LVEF) was 35%. Then, the diagnosis of TTS was initially confirmed.
Day 1	CCTA was performed, acute myocardial infarction was ruled out, and then the diagnosis of TTS was further confirmed.
Day 7	The patient was discharged in a stable condition.
1-year post-discharge	a repeat ECG was performed and demonstrated the disappearance of the previously observed Q waves and ST-T changes in the inferior and anterior wall leads. Concomitantly, a repeat echocardiogram indicated a significant improvement in left ventricular systolic function (LVEF = 61%).

The diagnostic conundrum intensifies when patients present with ST-segment elevation, creating an urgent need to differentiate TTC from acute coronary syndromes. Current guidelines emphasize coronary angiography as the gold standard for exclusion of obstructive disease (3). However, this invasive approach carries non-negligible risks for frail elderly patients, with complication rates exceeding 5% in octogenarians (4). Our case addresses this problem by demonstrating that stepwise noninvasive diagnosis of TTC can achieve diagnostic certainty while minimizing patient risk.

## Case description

**Figure 1:**
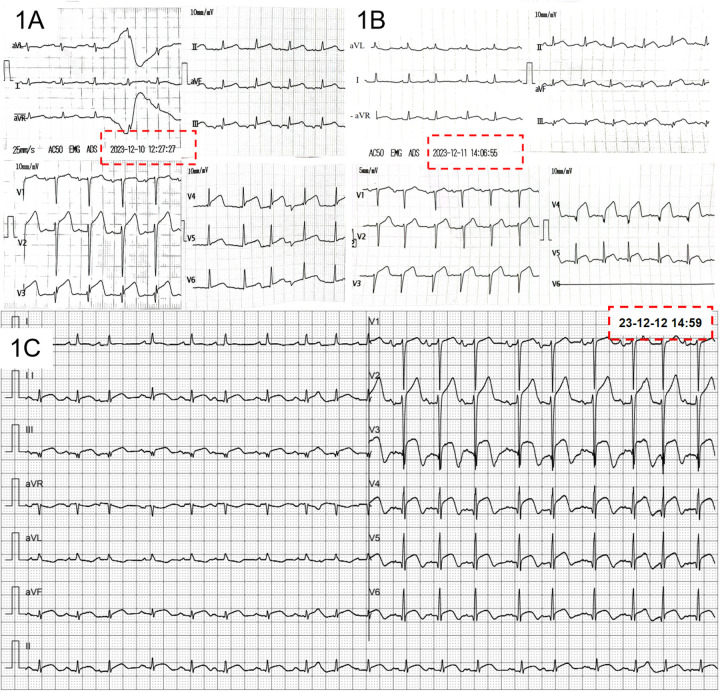
Initial ECG before admission **(A,B)** and at admission **(C)**.

An 88-year-old female patient with chest tightness and shortness of breath for 3 days was admitted to the hospital. She reported nausea (without vomiting or chest pain) triggered by emotional stress before admission. Symptoms exacerbated in the supine position. Electrocardiograms (ECG) were obtained on consecutive days from local community clinic, as depicted in Figures 1A,B. Subsequently, she was transferred to our hospital. Physical examination revealed a blood pressure of 112/73 mmHg, a body temperature of 36.3°C, a heart rate of 112 beats per minute, and a respiratory rate of 23 breaths per minute. Upon admission, a 12-lead ECG was performed (Figure 1C). Laboratory investigations indicated a serum hsTNI (high-sensitivity troponin I) level of 260.7 pg/ml (reference range 0–11.8 pg/ml) and an NT-proBNP (N-terminal pro–B-type natriuretic peptide) level of >35,000 pg/ml (reference range 0–450 pg/ml).

After admission, the patient was initially suspected to have Takotsubo cardiomyopathy (TTC) rather than acute anterior and inferior wall myocardial infarction. The primary basis for this consideration was the concurrent appearance of ST-T changes in the inferior wall (leads II, III, aVF) and anterior wall (leads V_3_—V_5_) on the ECG. The simultaneous ST-segment elevations in anterior and inferior leads localized the injury vector to the apex, consistent with apical involvement. In contrast, if the condition were an acute myocardial infarction caused by embolism in the proximal and middle segments of the anterior descending artery, ischemia and injury would predominantly manifest in the anterior wall, with the injury vector pointing anteriorly, and the ST-T changes in the inferior wall would typically be in the opposite direction.

**Figure 2:**
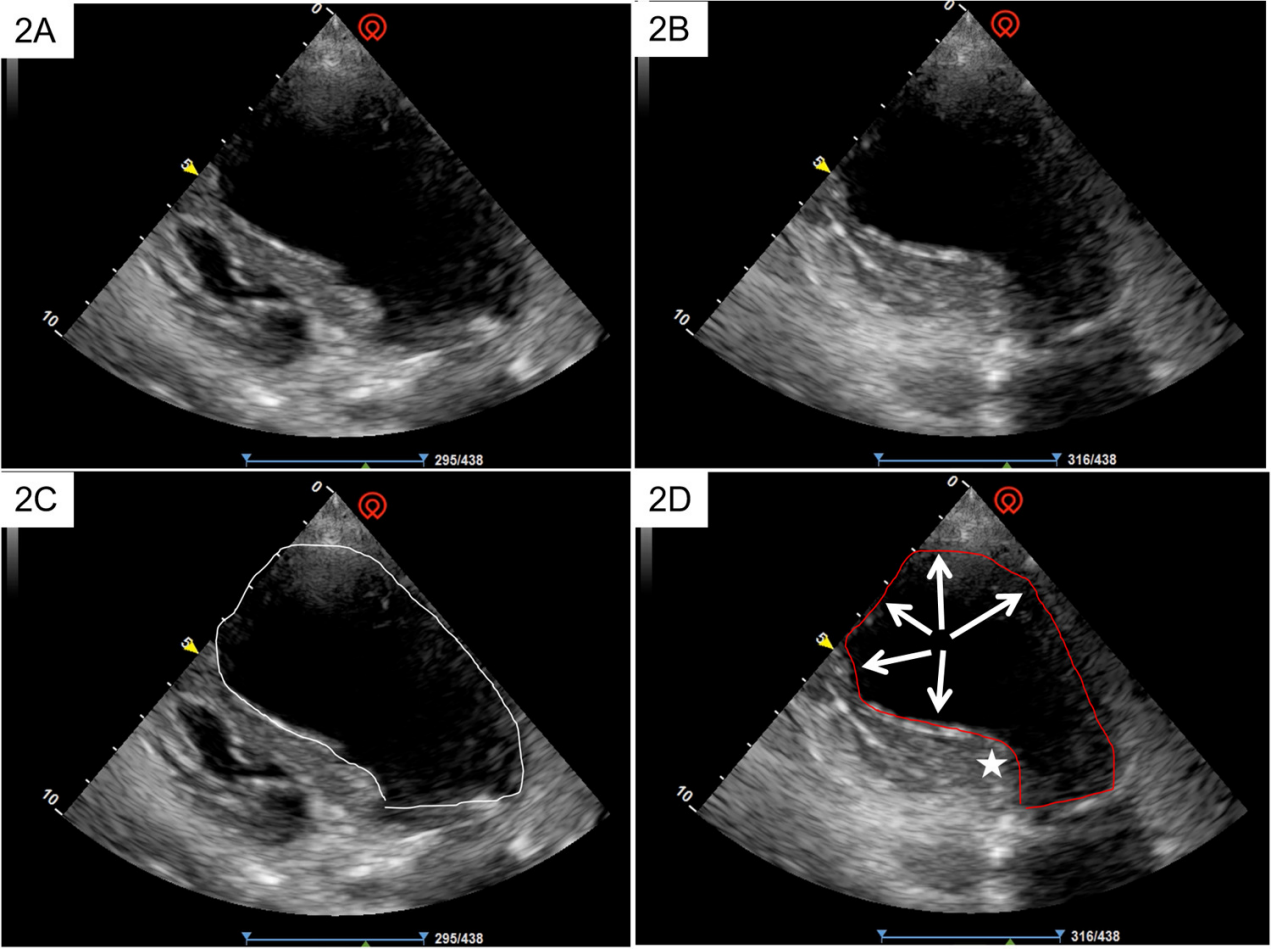
The echocardiographic manifestations of the long axis of the heart at the end of ventricular diastole **(A,C)**, and at the end of ventricular systole **(B,D)**. Comparative analysis demonstrated that only the basal portion of the ventricle exhibited contraction (indicated by the asterisk), whereas the apex scarcely contracted (indicated by the arrow).

Echocardiography was subsequently performed in half an hour, which demonstrated apical akinesis with preserved basal contractility. Figures 2A,C illustrate the echocardiographic manifestations of the long axis of the heart at the end of ventricular diastole, while Figures 2B,D depict those at the end of ventricular systole. Comparative analysis demonstrated that only the basal portion of the ventricle exhibited contraction (indicated by the asterisk), whereas the apex scarcely contracted (indicated by the arrow). The left ventricular ejection fraction (LVEF), as measured by the biplane Simpson method, was 35%. Supplementary echocardiographic videos of the long and short axes during cardiac pulsation are provided in the supplementary material for a more comprehensive assessment.

Taking into account the patient's advanced age and the low likelihood of severe coronary artery disease, a coronary computed tomography angiography (CCTA) was conducted instead of a coronary angiography (CAG), to delineate the coronary artery status while minimizing damage to the patient. The result of CCTA revealed a minimal amount of calcification and mild to moderate atherosclerosis in the coronary arteries (see Supplementary Material—images of CCTA). This finding effectively precluded the diagnosis of acute myocardial infarction (MI). Consequently, the diagnosis of TTC was further substantiated. The patient was discharged after comprehensive symptomatic and supportive treatment. Before discharge, the hsTNI level had decreased to 40.2 pg/ml, and the NT-proBNP level was 3,260 pg/ml.

**Figure 3:**
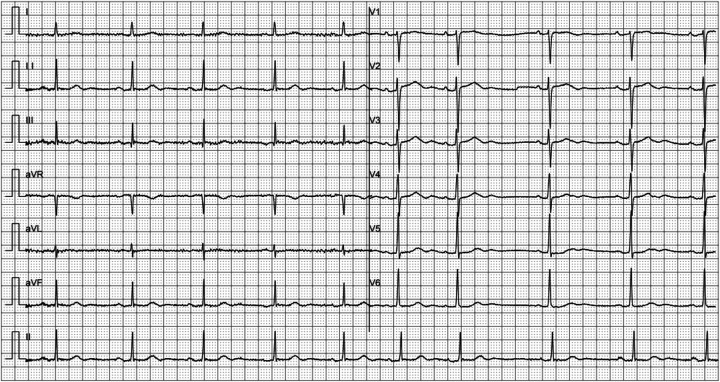
ECG follow-up at one year after first admission.

Surface ECG was performed at 12 months after the initial admission (Figure 3). The ECG demonstrated the disappearance of the previously observed Q waves and ST-T changes in the inferior and anterior wall leads. Concomitantly, a repeat echocardiogram indicated a significant improvement in left ventricular systolic function (61%).

## Discussion

TTC is characterized by systolic dysfunction predominantly localized to the left ventricular apex (81.7%), with less frequent involvement of the midventricular (14.6%), basal (2.2%), or focal (1.5%) regions (2). The prevailing etiological hypothesis suggests that TTC is triggered by a surge in circulating catecholamines and stress hormones, typically following exposure to physical or emotional stressors (2, 5). In this case, further inquiry into the patient's history revealed that three days prior to admission, her husband had passed away, leading to intense emotional fluctuations and, the development of TTC.

TTC represents a state of severe physical stress that can lead to an increase in catecholamine levels. This elevation activates the central autonomic nervous system, resulting in calcium overload within cardiac myocytes. The ensuing myocardial stunning and the precipitation of cardiomyopathy are the pathophysiological consequences. The electrocardiographic manifestations of TTC are relatively specific and include T-wave inversion, which is often profound and widespread, and the occurrence of negative T waves typically follows the resolution of the initial ST-segment elevation (6). Given that typical TTC predominantly affects the apex of the heart, the vectors of T-wave inversion or ST-segment elevation are directed towards the apex. According to the principles of electrocardiographic lead projections, this is manifested as the simultaneous presence of T-wave inversion and/or ST-segment elevation, and even the appearance of pathological Q waves, in the inferior and anterior wall leads. On an ECG, this finding is similar to what is seen in acute coronary syndrome (ACS). However, when a coronary angiography is performed, there is no evidence of acute plaque rupture or obstructive coronary artery stenosis. There are several well—recognized diagnostic criteria for TTC. The Heart Failure Association of the European Society of Cardiology (ESC), the Mayo Clinic Criteria, and the InterTAK Diagnostic Criteria are widely used in the medical field to diagnose TT C (3, 7, 8).

Recent technological advances have placed CCTA in the center of the non-invasive diagnostic workup of patients with CAD. The method was proven reliable in the diagnosis of relevant coronary artery stenosis. CCTA can identify different stages of the atherosclerotic process, including early atherosclerotic changes of the coronary vessel wall, a quality not met by other non-invasive tests (9). The CCTA's ability to differentiate plaque subtypes (calcified vs. non-calcified) strengthens its role in distinguishing stable CAD from lesions prone to rupture. This patient was found to have a mixed plaque in the proximal left anterior descending artery (LAD) with 43% stenosis and a calcified plaque in the mid-LAD with 55% stenosis. The CCTA excluded high-risk features, such as lipid-rich “napkin-ring” plaques, associated with Type I MI (10, 11).

Optimizing TTC diagnosis necessitates balancing accuracy and safety in elderly patients. Although current guidelines recommend invasive CAG to exclude obstructive disease, procedural risks (e.g., vascular complications, contrast nephropathy) may outweigh benefits in this population. In the present case, the convergence of ECG findings—ST-segment elevation in anterior/inferior leads followed by characteristic T-wave inversion—with ECG evidence of apical akinesia provided robust support for a provisional diagnosis of TTC. This multimodal approach supports CCTA as a safer alternative to invasive angiography, aligning with evolving clinical practices that prioritize noninvasive strategies when diagnostic confidence is high. Naturally, the efficacy of CCTA in diagnosing TTC still requires further clinical studies for validation, particularly in comparison to invasive CAG.

This case highlights the critical importance of ECG leads in diagnosis. The concurrent ST-T changes in inferior and anterior leads pointed to an apical lesion. Moreover, echocardiography was utilized to confirm the significant attenuation or disappearance of apical pulsation, and CCTA was applied to rule out acute myocardial infarction. This approach aligns with established diagnostic frameworks while exemplifying personalized medicine, wherein risk stratification informs the selection of minimally invasive modalities.

## Data Availability

The datasets presented in this article are not readily available because of ethical and privacy restrictions. Requests to access the datasets should be directed to the corresponding author.
