# Supplementary material for: Case Report: Stepwise noninvasive diagnosis of Takotsubo cardiomyopathy in an elderly patient–From ECG clues to echocardiographic and CTA confirmation
Source: Front Cardiovasc Med. 2025 Jun 4;12:1608992. doi: 10.3389/fcvm.2025.1608992 (PMC12174099; doi:10.3389/fcvm.2025.1608992)
Supplement: Supplementary file 1 [file Datasheet1.pdf]

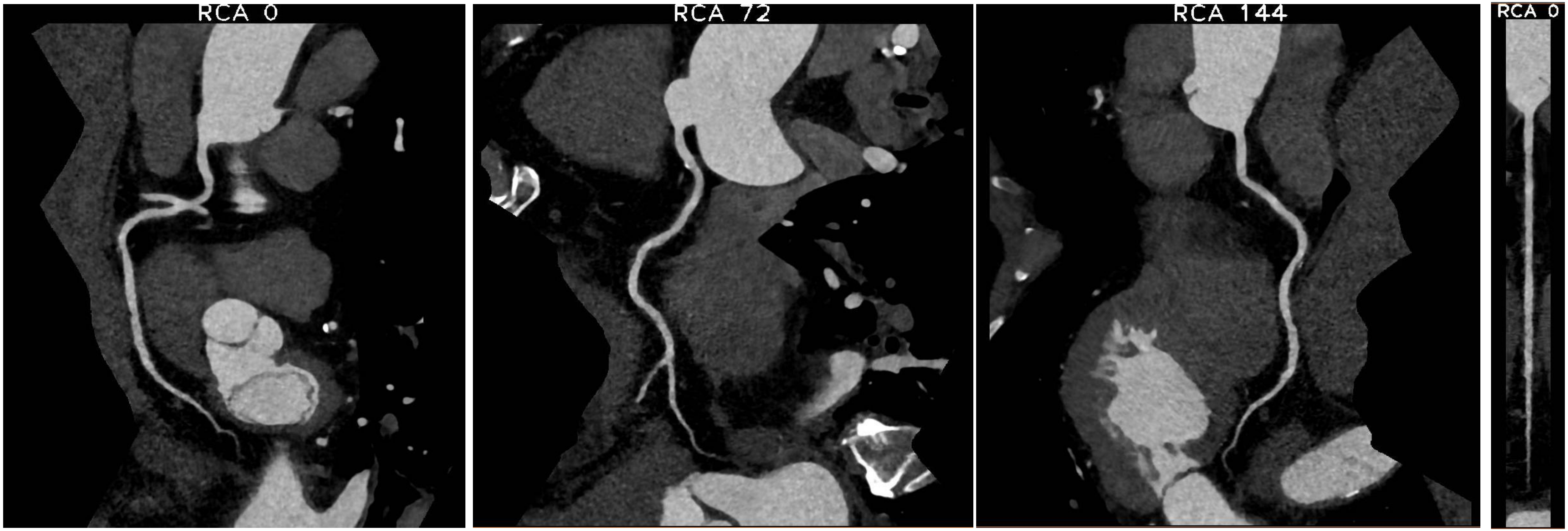
RCA 0
RCA 72
RCA 144
RCA 0

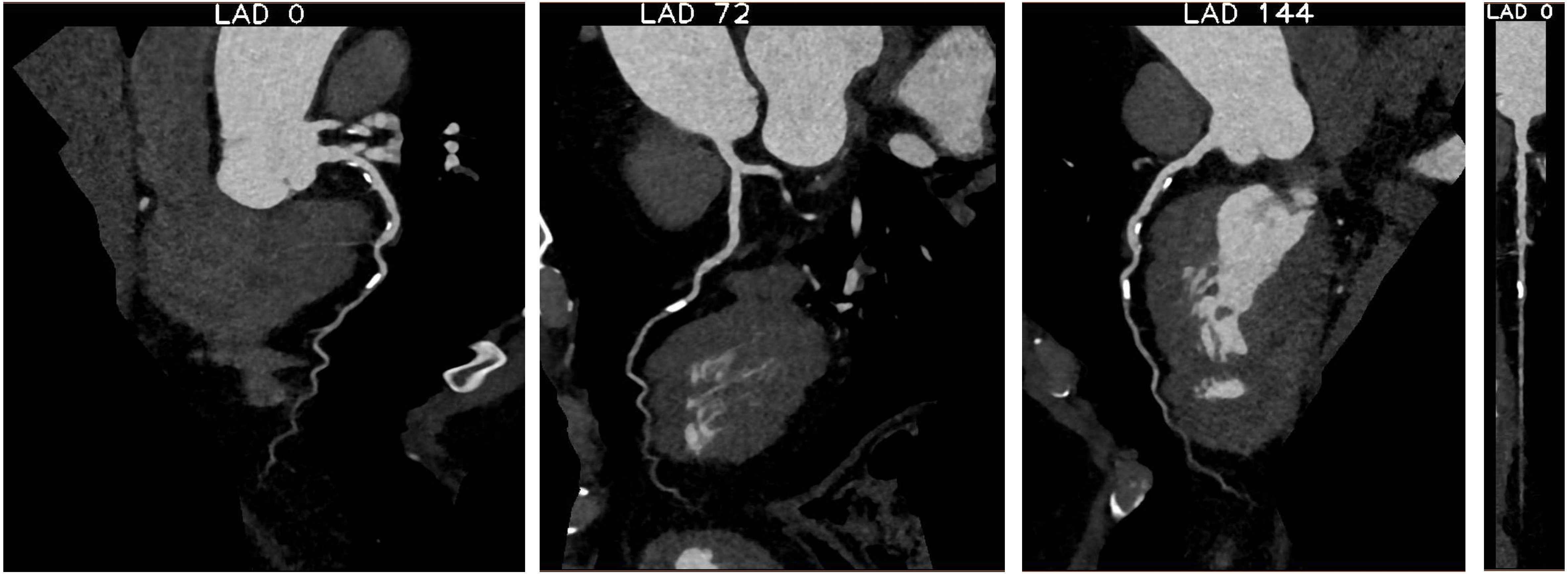
LAD 0
LAD 72
LAD 144
LAD 0

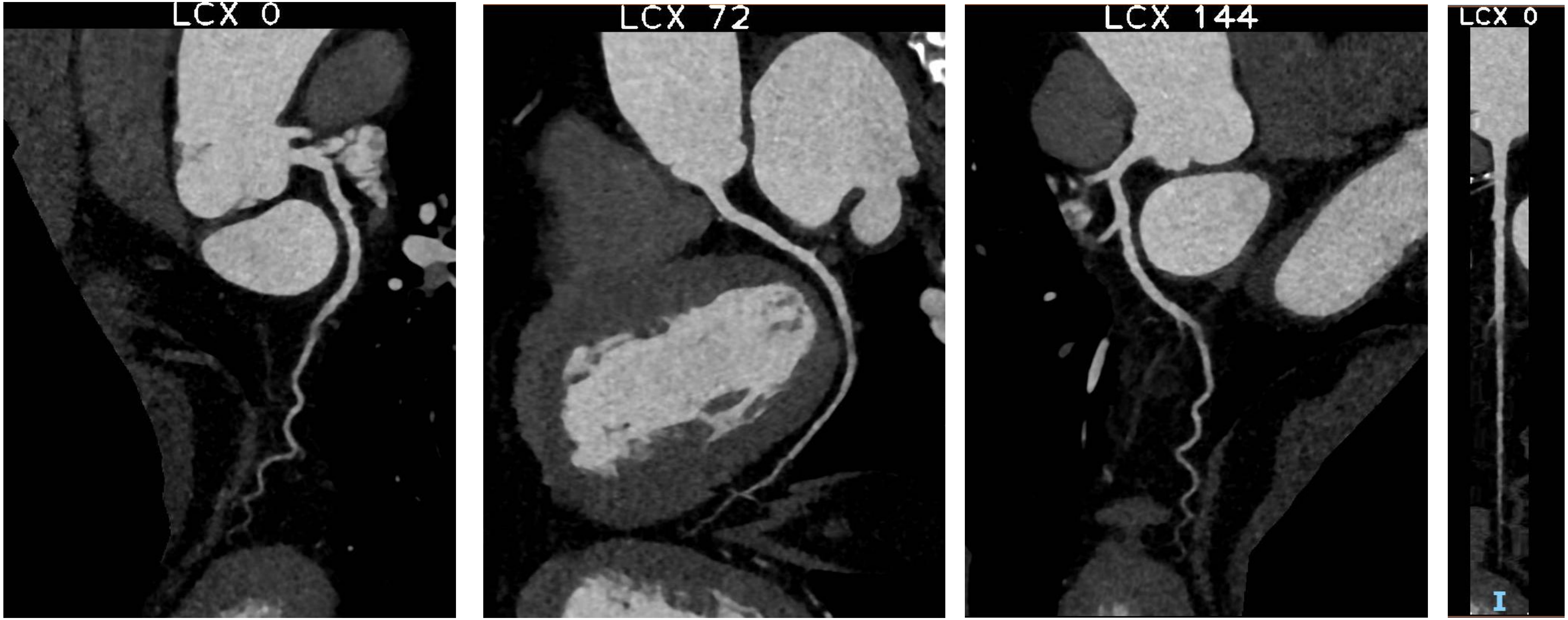
LCX 0
LCX 72
LCX 144
LCX 0
I

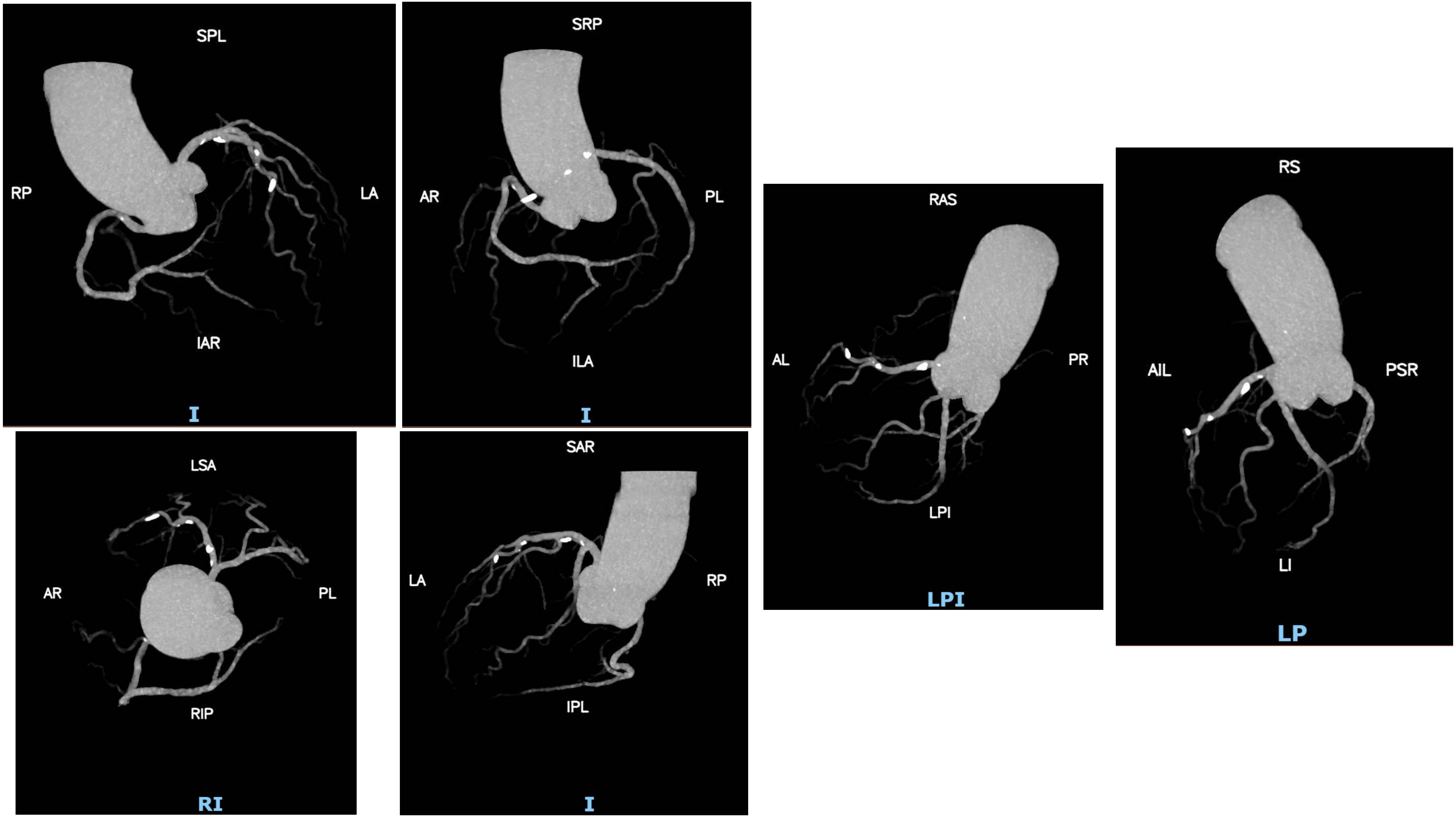
SPL
RP
LA
IAR
I
SRP
AR
PL
ILA
I
RAS
AL
PR
LPI
LPI
RS
AIL
PSR
LI
LP
LSA
AR
PL
RIP
RI
SAR
LA
RP
IPL
I

检查名称： 冠状动脉CTA

影像表现：

左主干（LM）钙化积分为0.00分。左前降支（LAD）钙化积分为223.80分。左回旋支（LCX）钙化积分为0.00分。右冠状动脉（RCA）钙化积分为20.95分。总钙化积分为244.75分。
冠状动脉呈右优势型。左主干起源于左窦，右冠状动脉起源于右窦。左主干(LM)未见明显狭窄。左前降支(LAD)近段管壁见混合斑块，管腔轻度狭窄(43%)；中段管壁见钙化斑块，管腔中度狭窄(55%)；远段未见明显狭窄。第一对角支(D1)、第二对角支(D2)未见明显狭窄。左回旋支(LCX)未见明显狭窄。第一钝缘支(OM1)、第二钝缘支(OM2)未见明显狭窄。中间支(RI)未见明显狭窄。右冠状动脉(RCA)近段管壁见钙化斑块，管腔轻微狭窄(20%)；中段、远段未见明显狭窄。右侧后降支(R-PDA)未见明显狭窄。左心室右后支(R-PLB)未见明显狭窄。

诊断意见：

左前降支(LAD)近段管壁见混合斑块，管腔轻度狭窄(43%)；中段管壁见钙化斑块，管腔中度狭窄(55%)。
右冠状动脉(RCA)近段管壁见钙化斑块，管腔轻微狭窄(20%)。
[CAD-RADS 3]

请结合临床，必要时DSA检查。

CTA Imaging Findings:

The calcium score of the left main coronary artery (LM) is 0.00. The calcium score of the left anterior descending artery (LAD) is 223.80. The calcium score of the left circumflex artery (LCX) is 0.00. The calcium score of the right coronary artery (RCA) is 20.95. The total calcium score is 244.75.

The coronary artery system is right-dominant. There is no significant stenosis in the left main coronary artery (LM). In the proximal segment of the left anterior descending artery (LAD), mixed plaques can be seen on the vessel wall, with mild stenosis of the lumen (43%); in the middle segment, there are calcified plaques on the vessel wall, causing moderate stenosis of the lumen (55%); and no significant stenosis is detected in the distal segment. The first diagonal branch (D1) and the second diagonal branch (D2) show no significant stenosis. The left circumflex artery (LCX) shows no significant stenosis. The first obtuse marginal branch (OM1) and the second obtuse marginal branch (OM2) show no significant stenosis. The ramus intermedius (RI) shows no significant stenosis. In the proximal segment of the right coronary artery (RCA), calcified plaques are present on the vessel wall, resulting in mild stenosis of the lumen (20%); no significant stenosis is observed in the middle and distal segments. The right posterior descending artery (R - PDA) shows no significant stenosis. The right posterolateral branch of the left ventricle (R - PLB) shows no significant stenosis.

Diagnostic Opinion:

In the proximal segment of the left anterior descending artery (LAD), there are mixed plaques on the vessel wall, with mild stenosis of the lumen (43%); in the middle segment, there are calcified plaques on the vessel wall, causing moderate stenosis of the lumen (55%). In the proximal segment of the right coronary artery (RCA), there are calcified plaques on the vessel wall, resulting in mild stenosis of the lumen (20%). [CAD - RADS 3]
